# Cross-Sectional Study of Atypical Swallowing and Occlusal Characteristics in 6–16-Year-Old Patients Presenting for Orthodontic Care

**DOI:** 10.3390/dj13120607

**Published:** 2025-12-17

**Authors:** Sara Caruso, Francesco Cipriani, Claudia Martino, Lucilla Calgani, Mauro Arcangeli, Roberto Gatto, Silvia Caruso, Antonella Mattei

**Affiliations:** 1 Department of Life, Health and Environmental Sciences, University of L’Aquila, 67100 L’Aquila, Italy; claudia.martino@student.univaq.it (C.M.); lucilla.calgani@univaq.it (L.C.); mauro.arcangeli@univaq.it (M.A.); roberto.gatto@univaq.it (R.G.); silvia.caruso@univaq.it (S.C.); antonella.mattei@univaq.it (A.M.); 2 Department of Molecular Medicine and Development, University of Siena, 53100 Siena, Italy; f.cipriani11@student.unisi.it

**Keywords:** atypical swallowing, malocclusion, temporomandibular joint (TMJ), dysfunction and muscle disorders, anterior open bite

## Abstract

**Introduction:** Malocclusion and dysfunctional or atypical swallowing are two conditions that significantly affect the health and well-being of the stomatognathic system, so much so that they often interact, influencing each other, and the presence of one can cause the onset or aggravation of the other. In this regard, over the years studies have been carried out that tried to discover the correlation between atypical swallowing and malocclusion. The aim is to evaluate the prevalence of dysfunctional swallowing in patients with malocclusion, to examine the pathophysiological mechanisms linking malocclusion and dysfunctional swallowing, and above all to investigate what potential risk factors may be. **Materials and Methods:** A sample of 60 patients aged between 6 and 16 years was analyzed at the Department of Dentistry of the University of L’Aquila. Some characteristics of the subjects’ face and posture were analyzed both from a frontal and lateral point of view. An orthodontic, temporomandibular joint, and masticatory muscle diagnosis was made. In addition, an examination of oral structures and functions was performed that allowed breathing, swallowing, chewing, and phono-articulation to be assessed. **Results:** It was observed that all the children had atypical swallowing, with significant postural abnormalities of the tongue; in fact, only 5% had a correct posture of the tongue at rest. In the analysis of occlusal characteristics, it emerged that with regard to the transverse plane, 21.67% of subjects have a condition of No Cross, while 10% show a Unilateral Cross. Finally, 68.33% show a Bilateral Cross. As far as the anterior–posterior plane is concerned, most of the subjects, equal to 76.67%, are placed in Class I, while 23.33% are in Class II. Finally, in relation to the vertical plane, 63.33% of subjects have normal occlusion, while 25% suffer from deep bite and 11.67% from open bite. The sample, stratified by presence or absence of alerts, shows significant differences for atypical swallowing (*p* = 0.031), for the presence of Class II malocclusion (*p* = 0.002), for low lingual posture, (*p* < 0.001), and for labial incompetence (*p* = 0.001). The multivariate logistic regression model showed that the presence of atypical swallowing (OR 1.04, 95% CI 1.04–1.07, *p* = 0.029), open bite malocclusion (OR 1.09, 95% CI 1.01–1.18, *p* = 0.013), low lingual posture (OR 1.11, 95% CI 1.04–1.18, *p* = 0.002), and the presence of labial incompetence (OR 1.06, 95% CI 1.02–1.10, *p* = 0.029) were significant clinical risk factors independently associated with the presence of alerts. **Conclusions:** The data collected confirm that atypical swallowing is a key element in the development of malocclusions, with a strong impact on posterior crossbite, anterior overjet, and other occlusal discrepancies. Among the data collected in the diagnostic phase, patients who presented at least one significant alert were also considered and atypical swallowing, low lingual posture, open bite malocclusion, and the presence of labial incompetence were statistically significant.

## 1. Introduction

Malocclusion and dysfunctional or atypical swallowing are two conditions that significantly affect the health and well-being of the stomatognathic system, so much so that they often interact, influencing each other, and the presence of one can cause the onset or aggravation of the other. However, the interconnection between malocclusion and dysfunctional swallowing is still the subject of debate and extensive research in the field of dentistry and speech therapy.

Atypical swallowing is a myofunctional alteration characterized by a postural change in the tongue during the swallowing process [1]. Atypical swallowing occurs when there is no transition from infant to adult-type swallowing and has a multifactorial etiology. The most common causes referred to are the following: finger sucking, onychophagia, mouth breathing, cheek chewing, dysmorphosis of various types of the stomatognathic system, muscle hypotonia, and diseases of the nervous system [2].

For many years, knowledge of the swallowing mechanism, its variants, and associated pathologies has become very important, as it is a problem with a very high incidence in the population. Clinical and epidemiological studies, in fact, suggest that its prevalence may be significant, with estimates ranging from 15% to 60% of the pediatric population [3]. 

This parafunction is often associated with malocclusions, in particular with skeletal problems such as open bite, ante-inclination of the maxillary plane, and post-inclination of the mandibular plane.

It is also often associated with dental problems such as diastema, protrusion of the upper incisors, increased overjet, and reduced overbite [4,5]. 

In this regard, over the years studies have been carried out that tried to discover the correlation between atypical swallowing and malocclusion

In 1946, Rix, studying a sample of 93 children between the ages of 7 and 12, found that 61 had atypical swallowing, and of these, 36 percent had malocclusion [6]. 

Later, in 1962, Werlich visited 640 elementary and middle school children. This study showed that 30.4% had dysfunctional swallowing; of these, 50.7% had a Class II Division 1 malocclusion and 98.5% had an open bite. In addition, in older children, he ascertained a significant relationship between atypical swallowing and posterior crossbite [7]. 

These studies have led to the conclusion of the possible existence of a two-way relationship between the two problems, suggesting a multidisciplinary approach, albeit with remaining uncertainties due to the lack of definitive data [7]. 

Understanding the connection between malocclusion and atypical swallowing is crucial as it impacts treatment decisions and helps develop a personalized treatment plan for patients that aims to correct both problems, reduce long-term complications such as temporomandibular joint (TMJ) dysfunction and muscle disorders, and improve patients’ quality of life [8].

The complexity of its origin and development, its wide diffusion in the population, and the connection with dento-skeletal problems make atypical swallowing a topic of considerable interest in the orthodontic and speech therapy field; therefore, it is still the subject of numerous debates, also because there is no work that evaluates this association by including only studies with adequate diagnostic methods for deviated swallowing [6]. 

The present study intends to examine their correlation with malocclusions, using valid diagnostic methods [1].

Specifically, it aims to evaluate the prevalence of dysfunctional swallowing in patients with malocclusion, to examine the pathophysiological mechanisms linking malocclusion and dysfunctional swallowing, to analyze the effect of malocclusion on swallowing habits and oral muscle function, and to determine which are the main characteristics or alerts present in the sample so as to represent risk factors.

## 2. Materials and Methods

### 2.1. Sample

The study was conducted according to the principles of the Declaration of Helsinki and received the approval of the Ethics Committee of the University of L’Aquila (Prot. N. 3752 of 04/02/2016).

A sample of 60 patients aged between 6 and 16 years was analyzed at the Dentistry Department of the University of L’Aquila.

Each subject was subjected to a careful anamnestic collection and a protocol for the detection of diagnostic elements for deviated swallowing.

The following inclusion criteria were applied for the selection of subjects: age between 6 and 16 years old; mixed dentition; and presence of malocclusion.

The only exclusion criterion was identified as a previous certificate of diagnosis of deviated swallowing.

### 2.2. Diagnostic Protocol

The diagnostic protocol is the result of various components that are listed below. General and facial observation: Some characteristics of the subject’s face and posture are analyzed both from a frontal and a lateral point of view.

Instrumental assessment of swallowing is a well-established and highly effective method for diagnosing and evaluating pathologies, as well as for planning rehabilitation interventions and achieving specific objectives with the help of an interdisciplinary team [9]. Instrumental measurements are essential to set up an appropriate therapy, monitor progress, and verify the achievement of the set goals [10]. The tools used for this assessment include the following.

- **Dynamometer:** This instrument is used to measure the resistance force of the lips during traction [11].

To take the measurement, a 20 mm diameter button connected to a cord is used, which is attached to the end of the instrument. The button is placed between the incisors and the lips and the force exerted by the lips is measured on the graduated scale of the instrument. According to Garliner, a normal value is between 3 and 5 pounds; higher or lower values may indicate hypotonia or hypertonia of the orbicularis muscle, respectively [11,12].

- **Myoscanner:** This electronic instrument is designed to measure the compressive force of the lips, the pressure exerted by the tongue when protruded, and the contraction of the masseter muscles [13]. The Myoscanner is equipped with an analog reader and a steel plate that detects the amount of force applied to the muscles being examined [11].
- **Wood’s lamp:** This tool is essential for diagnosing swallowing dysfunctions and alterations in lingual motility [12]. Used in the so-called “Payne Technique,” invented by Payne himself, Wood’s lamp uses fluorescence for analysis [14,15]. During the examination, fluoroscein is applied to the apex and edges of the tongue, which must be dried beforehand. The patient then swallows the saliva and, thanks to the ultraviolet light emitted by Wood’s lamp, the marks left by the fluoroscein inside the oral cavity are observed.

Orthodontic diagnosis: Within the context of the information, the orthodontic diagnosis compiled by the specialist approached by the subject is entered, so that it can be assessed whether the speech therapy intervention should be inserted before, during, or after the orthodontic one [8]. 

The orthodontic diagnosis was carried out through the collection of radiographic records (orthopantomography and teleradiography) and cephalometric analysis [16].

This is an indispensable tool for a complete and correct classification of the subject and flows into an evaluation protocol for the diagnosis of any deviated swallowing.

Mandible and masticatory muscles: These allow one to investigate resting posture, TMJ, and muscle control.

Tongue and masticatory muscles: This section analyzes the resting posture of the tongue, its appearance, lingual frenulum, and muscle lingual control.

Lips and labial muscles: This section investigates labial competence, labial frenulum, and muscle control of the lips.

Examination of oral structures and functions: This is the most specific part of the protocol as it examines the structures and functions that contribute to the formulation of the diagnosis. This section, in fact, allows one to evaluate breathing, swallowing, chewing, and phono-articulation.

### 2.3. Statistical Analysis

A descriptive analysis was conducted to describe the characteristics of the sample. Discrete and nominal variables were described with frequencies and percentages, and differences were assessed by the χ^2^ test or Fisher’s exact test, as appropriate. Quantitative variables were expressed as mean and standard deviations, and significance was assessed through the Mann–Whitney test.

The sample was dichotomized based on the presence or absence of alerts. In cases of statistical significance, the variables were included in a logistic regression model using backward stepwise selection to identify the factors associated with the presence of alerts. The dependent variable was the presence or absence of alerts, while the independent variables included sex, age, atypical swallowing, open bite malocclusion, low tongue posture, crowding, and lip incompetence. Associations were expressed as Odds Ratios (ORs), adjusted for age and sex in the model with 95% confidence interval (CI). The significance level was set at *p* < 0.05. Statistical analysis was performed using the STATA/BE 18.0 statistical package.

### 2.4. Results

This is a study on a representative sample of 60 children with a mean age of 9.24 (±2.09). Of these, 41.67% were boys (25 boys), while 58.33% were girls (35 girls) (Table 1). 

## 3. Results

As regards physiological history, several aspects related to birth and nutrition were evaluated. In total, 60% of babies were born by normal delivery, 26.67% by cesarean section, and 13.33% by emergency cesarean section. As for prolonged breastfeeding beyond six months, 40% received breastfeeding for more than six months, while 60% did not exceed this threshold. In addition, 46.67% of the infants were exclusively breastfed, 30% received mixed breastfeeding, and 23.33% were bottle-fed.

The time of weaning was also investigated: 11.67% of children were weaned by 4 months, 81.67% between 4 and 6 months, and only 6.67% after 6 months. Some eating and oral habits were observed. In total, 73.33% of children did not chew quickly, while 26.67% did.

With regard to dysfunctional oral habits, 91.67% did not practice finger sucking, while 8.33% did. However, 58.33% used a pacifier, and 71.67% used a bottle. In total, 66.67% did not use facilitating glasses to drink, and 91.67% did not have the habit of biting their lips. However, 28.33% showed behaviors such as biting nails, cuticles, or pencils.

In the remote pathological history, only 5% suffered from reflux.

Sleep quality and ENT problems were also investigated and it emerged that 16.67% suffered from bruxism (teeth grinding), 28.33% of children drooled during sleep, and 20% reported snoring. In total, 11.67% of children reported adenoid-related problems, while no cases of sleep apnea were reported.

In the analysis of occlusal characteristics, it emerged that with regard to the transverse plane, 21.67% of subjects have a condition of No Cross, while 10% show a Unilateral Cross. Finally, 68.33% show a Bilateral Cross. As far as the anterior–posterior plane is concerned, most of the subjects, equal to 76.67%, are placed in Class I, while 23.33% are in Class II. Finally, in relation to the vertical plane, 63.33% of subjects have a normal occlusion, while 25% suffer from deep bite and 11.67% from open bite.

It was observed that all the children had atypical swallowing, with significant postural abnormalities of the tongue; in fact, only 5% had a correct posture of the tongue at rest.

The sample, stratified by the presence or absence of alerts, shows significant differences for atypical swallowing (*p* = 0.031), for the presence of Class II malocclusion (*p* = 0.002), for low lingual posture (*p* < 0.001), and for labial incompetence (*p* = 0.001) (Table 2).

The multivariate logistic regression model showed that the presence of atypical swallowing (OR 1.04, 95% CI 1.04–1.07, *p* = 0.029), open bite malocclusion (OR 1.09, 95% CI 1.01–1.18, *p* = 0.013), low lingual posture (OR 1.11, 95% CI 1.04–1.18, *p* = 0.002), and labial incompetence (OR 1.06, 95% CI 1.02–1.10, *p* = 0.029) were significant clinical risk factors independently associated with the presence of alerts (Table 3). 

## 4. Discussion

The results of this study confirm the important role of atypical swallowing in the etiology of malocclusions; in particular, we find an association with posterior crossbite, deep bite, and anterior overjet.

The entire sample of children examined (100%) had atypical swallowing, showing a correlation with transverse, sagittal, and vertical occlusal problems. This is in line with the scientific literature, which identifies atypical swallowing and tongue thrust as crucial factors in the development of complex occlusal misalignments, such as posterior crossbite.

This study aims to evaluate whether swallowing alterations are associated with the type and severity of malocclusion.

The results regarding the prevalence of atypical swallowing confirm the data present in the literature, where a close correlation between atypical swallowing and malocclusions has been found, in particular with open bite [17,18]. However, contrary to what was reported by Osvenik et al. (2014) [19], who stated that atypical swallowing had the same prevalence in children up to three years of age regardless of the presence of a malocclusion, in our sample, atypical swallowing was observed in all subjects, showing a stronger relationship between the condition and malocclusions even in later ages.

Furthermore, while several studies suggest a strong association between open bite and atypical swallowing [18,20], in our study, only 11.67% of children had an open bite, with a majority presenting with a bilateral crossbite or deep bite condition.

In our sample, 41% of the children had a bilateral crossbite and 10% a unilateral crossbite, confirming the strong link between atypical swallowing and the development of transverse malocclusions.

Studies by Proffit and Melsen et al. [21,22] have shown that atypical swallowing, especially that associated with lingual thrust, has a significant impact on the development of these conditions, contributing to malocclusions.

In a study of 243 preschoolers (119 boys and 124 girls), the relationship between posterior crossbite, sucking habits, mouth breathing, and atypical swallowing was analyzed. The results show that at 3 years of age, the prevalence of atypical swallowing is similar between children with and without malocclusion, but in cases of crossbite, an increase in atypical swallowing is observed from 3 to 5 years of age, suggesting that the problem becomes more complex over time, as confirmed by the sample [23].

Posterior crossbite is one of the most frequent types of malocclusions during the deciduous and mixed dentition phase, with a prevalence of 7.2–23% [24]. 

It can cause mandibular asymmetry [25], affect the chewing and swallowing pattern [26,27], and reduce bite force [28].

The survey showed a higher frequency of immature swallowing in crossbite patients (50%) compared to the control group (26.67%). Occlusal interferences, common in patients with posterior crossbite, may lead to a compensatory position of the tongue between the teeth to stabilize the mandible and reduce pain, suggesting a possible correlation between immature swallowing and posterior crossbite etiology. The lingual thrust during swallowing reduces the pressure against the jaw while increasing that of the perioral and buccinator muscles against the maxillary arch, thus slowing down the normal transverse development of the jaw [21,22].

Lingual thrust was found to be a determining factor in our sample, with 95% of children presenting with inadequate lingual posture at rest. Some researchers have suggested that protrusion of the tip of the tongue during swallowing is associated with AOB (anterior open bite); however, other researchers have suggested that this protrusion is the result of functional adaptation of an existing malocclusion [29].

A case–control study was conducted to determine the association between different variables and the presence of AOB.

Factors such as the position of the tongue during swallowing, phoniatric changes, and the size of the dental arch in children with AOB or NVO (normal vertical overbite) were evaluated. Sprains caused by tongue thrust (tongue contact with the lingual surface of the upper incisors), tongue protrusion during swallowing (tongue protruding between the upper and lower teeth), inferior intermolar distance, upper total length, upper perimeter, and posterior depth were considered responsible for 64.6% of cases of AOB and were correctly predicted in 83.8% of cases in the study population. These results suggest that the position of the tongue at rest and the position of the tongue during function define the determination of altered occlusal patterns such as those seen in anterior open bite [24,30,31]. 

These findings are supported by the results of a study conducted by Pedrazzi et al. [32], who found that the tongue is the primary cause of the development and perpetuation of anterior open bite, and by studies conducted by Maspero et al. [33] and Gonçalves FM et al. [6], who described abnormal swallowing as a risk factor for occlusal changes such as anterior open bite, posterior crossbite, and proclination of the incisors.

In accordance with these results, our study presents, as statistically significant alert factors present in our open bite malocclusion sample (OR 1.09, 95% CI 1.01–1.18, *p* = 0.013), atypical swallowing (OR 1.04, 95% CI 1.04–1.07, *p* = 0.029), as well as lip incompetence (OR 1.06, 95% CI 1.02–1.10, *p* = 0.029) and low lingual posture OR 1.11, 95% CI 1.04–1.18, *p* = 0.002) which consequently create breathing problems, as we know from studies in the literature that the correct respiratory function (nasal breathing) requires the lip seal and the tongue in contact with the palate, so as to allow proper craniofacial growth and development [34].

## 5. Conclusions

The data collected state that atypical swallowing is a key element in the development of malocclusions, with a strong impact on posterior crossbite, anterior overjet, and other occlusal discrepancies. Lingual thrust and postural changes of the tongue, together with dysfunctional oral habits and respiratory dysfunctions, are the main factors contributing to the development of occlusal misalignments in the analyzed sample. These data underscore the importance of early diagnosis and timely intervention to correct dysfunctional oral habits and improve lingual posture. As evidenced by previous studies, early treatment of malocclusions, such as posterior crossbite, along with correction of atypical swallowing, can lead to better long-term outcomes [34,35]. A limitation of this study is its observational nature and the relatively small sample of subjects. In addition, the lack of a control group limited the possibility of comparing the results with children without malocclusions or with normal oral habits. As other research has shown, a more rigorous study with proper controls and longitudinal analyses could provide greater certainty of the evidence.

## Figures and Tables

**Table 1:** Demographic characteristics (*n* = 60).

Sex, *n* (%)	
Males	25 (41.67)
Females	35 (58.33)
Child age in years, media ± DS	9.24 ± 2.09
Anamnesis (*n* = 60)	
Type of delivery, *n* (%)	
Normal	36 (60.00)
Cesarean	16 (26.67)
Emergency	8 (13.33)
Breastfeeding > 6 months	
No	36 (60.00)
Yes	24 (40.00)
Type of breastfeeding	
Breast	28 (46.67)
Mixed	18 (30.00)
Artificial	14 (23.33)
Weaning	
Up to 4 months	7 (11.67)
4 to 6 months	49 (81.67)
>6 months	4 (6.67)
Chew quickly	
No	44 (73.33)
Yes	16 (26.67)
Reflux	
No	57 (95.00)
Yes	3 (5.00)
Finger sucking	
No	55 (91.67)
Yes	5 (8.33)
Pacifier	
No	25 (41.67)
Yes	35 (58.33)
**Feeding bottle** No Yes	17 (28.33) 43 (71.67)
Facilitating glasses No Yes	40 (66.67) 20 (33.33)
Bites lips No Yes	55 (91.67) 5 (8.33)
Bites nails, cuticles, pencils No Yes	43 (71.67) 17 (28.33)
Bruxism No Yes	50 (83.33) 10 (16.67)
Drools during sleep No Yes	43 (71.67) 17 (28.33)
Snoring No Yes	48 (80.00) 12 (20.00)
Adenoids No Yes	53 (88.33) 7 (11.67)
Freediving No Yes	60 (100.00)
Facilities	
Head Aligned Prefixed Inclined	19 (31.67) 26 (43.33) 15 (25.00)
Asymmetrical shoulders No Yes	46 (76.67) 14 (23.33)
Dorsal kyphosis Physiological Accentuated	37 (61.67) 23 (38.33)
Lumbar lordosis Physiological Accentuated	45 (75.00) 15 (25.00)
Narrow nostrils No Yes	49 (81.67) 11 (18.33)
Cheeks Pronounced cheekbone Fat Sagging	31 (51.67) 16 (26.67) 13 (21.67)
Dark circles No Yes	30 (50.00) 30 (50.00)
Transverse plane No cross Cross monolateral Cross bilateral	13 (21.67) 6 (10.00) 41 (68.33)
Anterior–posterior plane Class I Class II	46 (76.67) 14 (23.33)
Vertical plane Normal Deep bite Open bite	38 (63.33) 15 (25.00) 7 (11.67)
Mandibular opening movement Adequate Inadequate	58 (96.67) 2 (3.33)
Mandibular closure movement Adequate Inadequate	54 (90.00) 6 (10.00)
Mandibular protrusion movement Adequate Inadequate	49 (81.67) 11 (18.33)
Mandibular lateralization movement Adequate Inadequate	46 (76.67) 14 (23.33)
Lingual posture at rest Adequate Inadequate	3 (5.00) 57 (95.00)
Tongue Tonic Altered	38 (63.33) 22 (36.67)
Lingual frenulum Normal Altered	45 (75.00) 15 (25.00)
Lingual protrusion movement Adequate Inadequate	34 (56.67) 26 (43.33)
Lingual elevation movement Adequate Inadequate	29 (48.33) 31 (51.67)
Tongue retraction and lowering movement Adequate Inadequate	45 (75.00) 15 (25.00)
Lip incompetence No Yes	36 (60.00) 24 (40.00)
Lacing musculature: internal orbicularis Adequate Inadequate	39 (65.00) 21 (35.00)
Lip muscles: orbicularis “O” Adequate Inadequate	25 (41.67) 35 (58.33)
Lip muscles: buccinator Adequate Inadequate	20 (33.33) 40 (66.67)
Functions	
Respiration Nasal Oronasal Oral	37 (61.67) 14 (23.33) 9 (15.00)
Deglutition Typical Atypical	60 (100.00)
Mastication Adequate Inadequate	40 (66.67) 20 (33.33)
Voice quality alterations	
No	45 (75.00)
Yes	15 (25.00)
Saliva control	
Present	57 (95.00)
Absent	3 (5.00)
**Dyslalia**	
No	34 (56.67)
Yes	26 (43.33)

**Table 2:** Descriptive analysis of the characteristics of the sample, stratified by presence or absence of alerts.

		Facilities		
	Total *N* = 60	No Alerts *n* (%) 15 (25.00)	Presence of at Least One Alert *n* (%) 45 (75.00)	*p*-Value
Age, media ± DS	9.24 ± 2.09	8.65 ± 1.81	9.44 ± 2.13	0.236 *
Sex, *n* (%)				0.232 **
Males	25(41.67)	4 (26.67)	21 (46.67)	
Females	35 (58.33)	11 (73.33)	24 (53.33)	
Atypical swallowing, media ± DS				
Dysfunctionality, %	39.62 ± 24.68	27.01 ± 11.65	43.82 ± 26.48	<0.031 **
Open bite malocclusion, media ± DS				
Dysfunctionality, %	13.00 ± 13.00	5.37 ± 10.52	15.54 ± 12.84	0.002 **
Low lingual posture, media ± DS				
Dysfunctionality, %	31.68 ± 25.61	8.90 ± 8.62	39.27 ± 24.91	<0.001 **
Crowding, media ± DS				
Dysfunctionality, %	29.58 ± 18.69	20.83 ± 7.72	32.5 ± 20.37	0.08 **
Lip incompetence, media ± DS				
Dysfunctionality, %	32.93 ± 19.77	19.59 ± 12.82	37.37 ± 19.78	0.001 **

* Wilcoxon rank-sum test (Mann–Whitney); ** Fisher’s exact test.

**Table 3:** Factors associated with presence of at least one alert.

Factors	OR°	IC 95%	*p*-Value
Atypical swallowing	1.04	1.04–1.07	0.029
Open bite and malocclusion	1.09	1.01–1.18	0.013
Low lingual posture	1.11	1.04–1.18	0.002
Lip incompetence	1.06	1.02–1.10	0.005

**OR°** Adjusted for age and sex; *p* < 0.05, statistically significant difference between presence/absence of alert.

## Data Availability

The raw data supporting the conclusions of this article will be made available by the authors on request.
